# Fabrication and Characterization of New Composite Tio_2_ Carbon Nanofiber Anodic Catalyst Support for Direct Methanol Fuel Cell via Electrospinning Method

**DOI:** 10.1186/s11671-017-2379-z

**Published:** 2017-12-06

**Authors:** N. Abdullah, S. K. Kamarudin, L. K. Shyuan, N. A. Karim

**Affiliations:** 1 Fuel Cell Institute, Universiti Kebangsaan Malaysia, 43600 Bangi, Selangor Malaysia; 2 Department of Chemical and Process Engineering, Universiti Kebangsaan Malaysia (UKM), 43600 Bangi, Selangor Malaysia

**Keywords:** Carbon nanofiber, Electrospinning, Direct methanol fuel cell (DMFC), Catalyst support

## Abstract

Platinum (Pt) is the common catalyst used in a direct methanol fuel cell (DMFC). However, Pt can lead towards catalyst poisoning by carbonaceous species, thus reduces the performance of DMFC. Thus, this study focuses on the fabrication of a new composite TiO_2_ carbon nanofiber anodic catalyst support for direct methanol fuel cells (DMFCs) via electrospinning technique. The distance between the tip and the collector (DTC) and the flow rate were examined as influencing parameters in the electrospinning technique. To ensure that the best catalytic material is fabricated, the nanofiber underwent several characterizations and electrochemical tests, including FTIR, XRD, FESEM, TEM, and cyclic voltammetry. The results show that D18, fabricated with a flow rate of 0.1 mLhr^−1^ and DTC of 18 cm, is an ultrafine nanofiber with the smallest average diameter, 136.73 ± 39.56 nm. It presented the highest catalyst activity and electrochemical active surface area value as 274.72 mAmg^−1^ and 226.75m^2^ g^−1^_PtRu_, respectively, compared with the other samples.

## Background

Direct methanol fuel cell (DMFC) is one of the future renewable power-generating systems and very environmentally friendly. The system generates electrical energy using a liquid fuel (methanol) directly without any additional devices or combustion processes. The advantages of DMFCs are their simplicity, high specific energy, low operating temperature, and easy start-up with instant refueling [1]. However, DMFC systems still suffer from several limitations, such as catalyst poisoning and slow reaction kinetics, which lead to the system having low performance and power output [2]. Both of these limitations are due to the catalyst and material used in this system.

Platinum (Pt) is the common catalyst used in DMFC. However, Pt can lead towards catalyst poisoning by carbonaceous species, thus reduces the performance of DMFC. Later, platinum-ruthenium (PtRu) is introduced to increase the reaction rate, but the kinetic parameter of the catalyst is still the one of a major problem in DMFC. Therefore, the alteration towards this bimetallic catalyst starts to get placed in the field of DMFC catalyst. One of the most attractive approaches among researcher is introducing the metal oxide and nanomaterials as the side-catalyst component. Titanium dioxide (TiO_2_) is a metal oxide that is gaining a lot of attention from research developer. TiO_2_ has various beneficial properties, which is non-toxic, non-flammable, and highly resistant to corrosion [1], can increase the electrochemical and thermal stability [3], and affect the electronic properties and bifunctional mechanism of composite catalysts [4]. Ito et al. [5] developed PtRu/TiO_2_-embedded carbon nanofiber (CNF) (PtRu/TECNF), and Ercelik et al. [6] presented the PtRu/C-TiO_2_ as an electrocatalyst in DMFC application, and the result shows that the performance of this new composite electrocatalyst is higher than PtRu catalyst.

Nanomaterial is one of the nanotechnologies that fascinated in a wide range of application including energy conversion. There are numerous types of nanomaterials in the energy conversion field, which are nanofibers, nanotubes, nanowires, nanorods, and others. This material becomes the main attraction in energy material research because of the dimensional reduction to the nanometer scale that can affect many elementary steps, including charge transfer and molecular rearrangement, as well as the surface properties to provide high interfacial volume fractions and enhanced reaction rates [7]. This study focuses on nanofiber structures for both materials, TiO_2_ metal oxide and carbon. This is due to the special properties of nanofibers that can provide high surface/volume and aspect ratios [7], high electrical conductivity, good mechanical strength, and uniform dispersion of catalyst, which can increase the electrocatalytic activity [8].

**Fig. 1:**
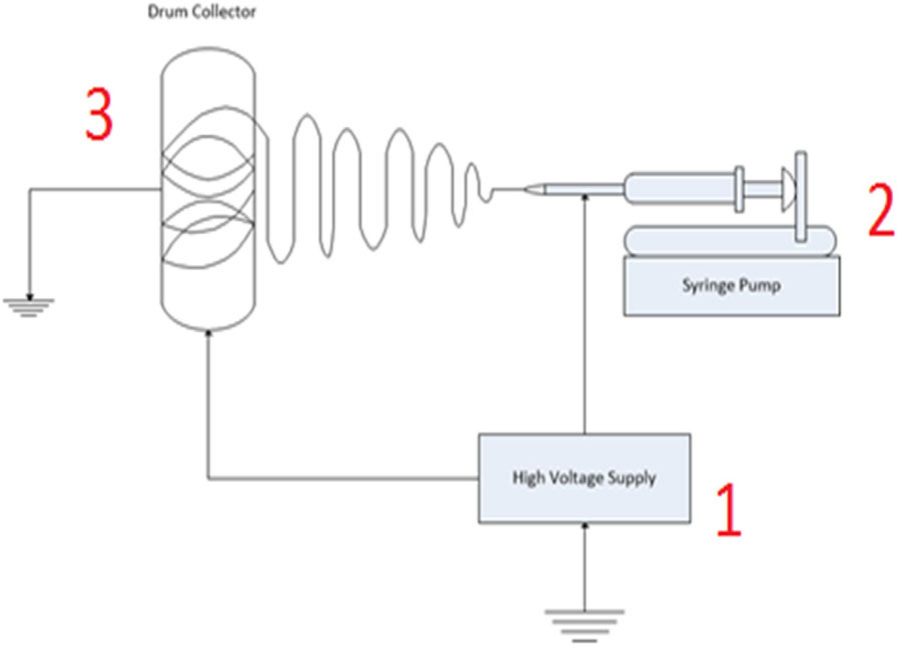
Electrospinning set up for all main components

Nanofibers can be produced by several processes, including melt blowing, interfacial polymerization, electrospinning, and antisolvent-induced polymer precipitation [9]. Recently, electrospinning is the main choice among researchers due to the great benefit of producing ultrafine nanofiber structures. Electrospinning is a unique process for the formation of fibers with submicron-scale diameters (in the nanometer to micrometer range) using polymer-based solutions or melts through electrostatic forces [10]. There are three main components for electrospinning: a high voltage power supply (several tens of kVs), the spinneret (syringe with a needle), and a grounded collector (e.g., plate or rotating collector) [11, 12]. Figure 1 illustrates the overall process and set up for the electrospinning process. Therefore, electrospinning is popular due to its simple procedure, versatile, high-yield, effective, and having a more economical process [7, 13].

This process has several parameters that can be tuned in order to obtain the optimal nanofiber structures, either for diameter or surface morphology, and the influencing parameters are different for each material. The parameters can be divided into three main categories: solution, ambient, and process parameters. This study is focused on process parameters, and solution flow rate and distance between the needle tip and the collector (DTC) were chosen as the main influencing parameters to obtain the smallest diameter. This is due to the small amount of research focused on these parameters [14], even though they have been considered as main variables for obtaining ultrafine nanofibers [15–18].

Thus, this study presents the composite TiO_2_ carbon nanofiber as catalyst support on the anode electrode. This combination of the composite is expected to increase the electrocatalytic activity and lowering the catalyst poisoning in order to boost the overall performance of DMFC. The main objectives of this study are to fabricate the smallest possible of nanofiber diameter to increase the surface area and provide more active spot for catalytic reaction and enhance the DMFC performance. The fabrication of nanofibers involves several steps, including sol-gel, electrospinning, stabilization, and carbonization processes. To obtain the smallest diameter nanofibers, the electrospinning parameters of flow rate and DTC are taken as the main variables in this study. The prepared nanofibers are characterized by Fourier transform infrared (FTIR) spectroscopy, X-ray diffraction (XRD), and scanning electron microscopy (FESEM). All the catalyst supports with different electrospinning parameter are deposited on PtRu (PtRu/TiO_2_-CNF) and evaluated by electrochemical active surface area (ECSA) analysis and cyclic voltammetry (CV) to evaluate the performance and determine their potential as catalyst supports in DMFCs. The experimental results show the effect of the electrospinning parameters on the nanofiber diameter, as well as their potential in DMFC applications.

## Methods/Experimental

### Materials

Poly(vinyl acetate) (PVAc, Mw = 500,000), dimethylformamide (DMF, 99.8%), titanium isopropoxide (TiPP, 97% content), acetic acid (99.7%), and Ru precursor (45–55% content) were obtained from Sigma-Aldrich Co., Ltd., while Pt precursor (40% content) and ethanol (99.8%) was received from Merck, Germany and R&M Chemical Reagents, respectively. All chemicals were used without any further purification. The main apparatus, electrospinning machine, is branded with Nfiber N1000, Progene Link Sdn. Bhd., and ultrasonic cell crusher INS-650Y is from INS Equipments Trading Co., Ltd., China.

### Preparation of TiO_2_-CNF Nanofibers

The sol-gel method begins with the preparation of a polymer solution, where PVAc (11.5 wt%), as the carbon source, was dissolved in the solvent, DMF. The polymer solution was stirred at 60 °C for 1 h and then stirred overnight at room temperature. The TiO_2_ precursor, TiPP, and polymer solution were mixed in a 1:1 ratio, and a small amount of acetic acid and ethanol was added to the polymer solution. The mixture was homogenized by an ultrasonic cell crusher for 60 s. Then, the solution was transferred to a syringe for injection in a nanofiber electrospinning unit. The applied voltage was 16 kV, while the flow rate and DTC were manipulated in the range of 0.1–0.9 mLh^−1^ and 14–18 cm. The flow rate was set at 0.1, 0.5^,^ and 0.9 mLh^−1^, denoted F0.1, F0.5 and F0.9, respectively. The samples with DTC values of 14, 16, and 18 cm are denoted D14, D16, and D18, respectively. The fabricated nanofiber was rested for 5 h at room temperature before being stabilized for 8 h at 130 °C. The stabilized nanofiber was carbonized at 600 °C for 2 h under a nitrogen atmosphere using a tube furnace and then crushed by mortar and pestle for 5 min before further use in this study. The mass loading for all samples is the same, which is 6.67 mgs^−1^.

### Deposition of Catalyst

The TiO_2_-CNF nanofibers were added into a mixture of isopropyl alcohol (IPA) and deionized water (DI water) and sonicated in an ultrasonic bath for 20 min. The precursor of the platinum and ruthenium catalyst (20 wt% with 1:1 ratio) was mixed into the solution and stirred for 20 min. Then, the pH of the mixed solution was adjusted with NaOH solution until reaching pH 8. The temperature was raised to 80 °C, and 25 ml of 0.2 M NaBH_4_ was added dropwise into the mixed solution. The solution was stirred for another 1 h. The mixture was then cooled, filtered, and washed repeatedly. The catalyst powder was dried at 120 °C for 3 h and finally crushed using a mortar and pestle to obtain a fine catalyst powder that was ready for use in the performance tests.

### Characterization of the Catalyst

The chemical compound in the catalyst support was identified using Fourier transform infrared spectroscopy (FTIR, PerkinElmer), and X-ray diffraction (XRD, D8 Advance/Bruker AXS, Germany) was used to analyze the pattern and crystal structure of the samples. The morphology and size distribution of the samples were analyzed by field emission scanning electron microscopy (FESEM, SUPRA 55VP). Transmission electron microscopy (TEM, Tecnai G2 F20 X-Twin) was used to observe the detailed structure and elemental distribution of the nanofibers.

### Evaluation of the Electrochemical Measurement

The performance was measured for all catalysts fabricated with different parameters. The PtRu catalyst was deposited on the TiO_2_-CNF catalyst support for evaluation by electrochemical measurements. These measurements were obtained using a three-electrode cell system, which uses cyclic voltammetry (CV) to examine the catalyst activity in the methanol oxidation reaction (MOR) using an Autolab electrochemical workstation. The three-electrode cell system was operated at room temperature and involved a Pt, silver/silver chloride (Ag/AgCl), and glassy carbon electrode (GCE, 3 mm diameter) as the counter, reference, and working electrode. Before starting the measurement, the GCE was cleaned with alumina and polishing paper, tracing a rounded pattern resembling the number “eight,” several times. Then, the GCE was rinsed with DI water and sonicated for 30 s before use. The catalyst ink for the GCE was prepared by dispersing 15 mg of catalyst into a mixture of 400 μl DI water, 400 μl IPA, and 125 μl Nafion solution (5 wt%) for 30 min. Then, 2.5 μl of catalyst ink was coated onto the GCE using a micropipette and dried for 1 h at room temperature before being heated at 80 °C for another 30 min. The electrolyte was a solution of 0.5 M H_2_SO_4_ in 2 M methanol, and it was bubbled for 20 min with nitrogen gas to remove any oxygen. The CV measurement was performed over a potential range of − 0.1–1.1 V vs. Ag/AgCl at a scan rate of 50 mVs^−1^.

## Results and Discussion

### Structural Characterization

#### Effect of Flow Rate

**Fig. 2:**
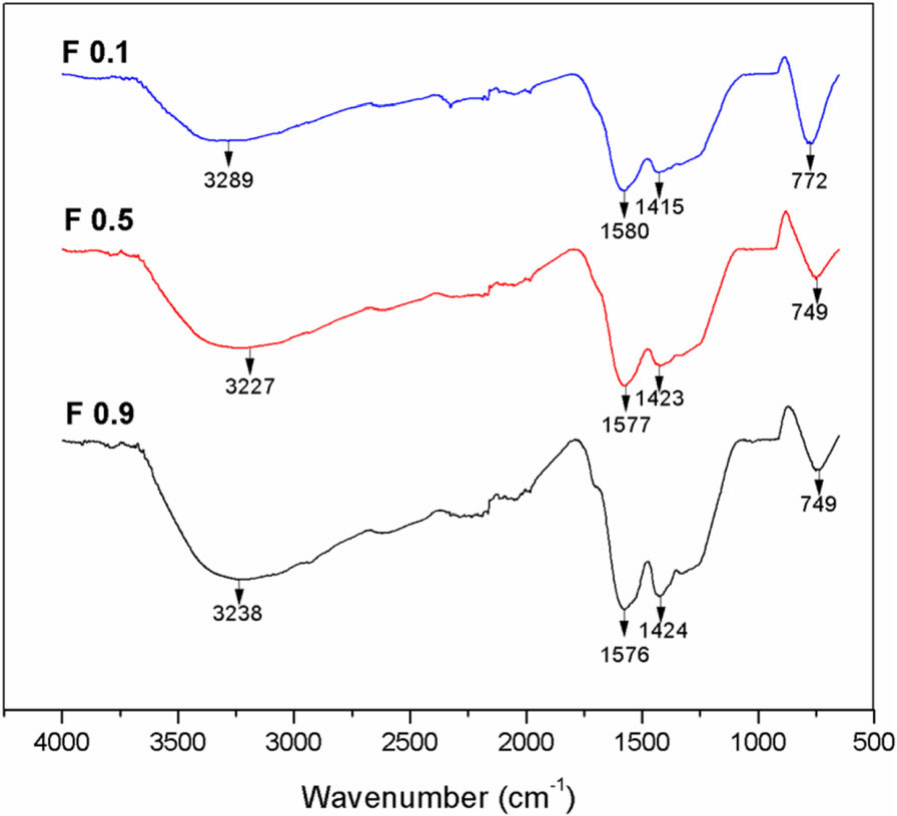
The IR spectrums for the TiO_2_-CNF sample with different flow rate parameter at the range of 650 to 4000 cm^−1^ wavenumber

FTIR spectroscopy was performed on the TiO_2_-CNF samples to identify the present chemical compounds. The IR spectra of the samples produced at different flow rates are shown in Fig. 2. The spectra revealed chemical bonding signals representative of TiO_2_ and carbon. The medium and broad peaks at 3200–3600 cm^−1^ represent O-H functional groups, while the sharp and strong C=O absorption band was located in the region of 1550–1850 cm^−1^ [19]. Peaks for alkanes (C-H groups) are weak and broad and located in the regions of 1300–1450 cm^−1^. However, C-C groups supposedly appear at very low wavenumbers, below 500 cm^−1^ [19] and do not exist in the spectra due to the small range of wavenumber (4000 cm^−1^ < wavenumber > 50 cm^−1^) produce by the spectrum. The medium and sharp bands in the region of 650–900 cm^−1^ belong to Ti-O groups, as suggested by Ding et al. [20]. The IR spectra feature all the functional groups in the TiO_2_-CNF samples. All samples have similar wavenumbers and peaks, which indicates that the flow rate of the polymer solution during electrospinning does not affect the chemical compounds in the sample.

**Fig. 3:**
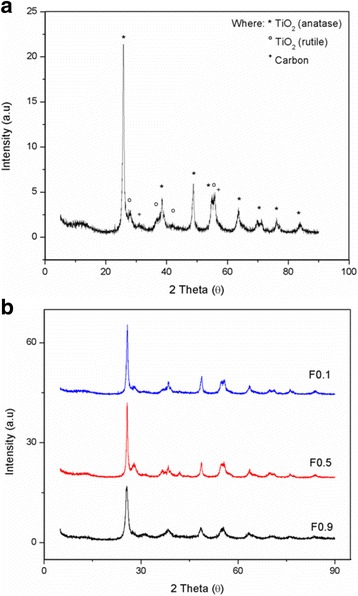
X-ray diffraction patterns of **a** individual TiO_2_-CNF sample and **b** different flow rate sample

The TiO_2_-CNF samples fabricated at flow rates of 0.1, 0.5^,^ and 0.9 mLh^−1^ are denoted F0.1, F0.5 and F0.9, respectively. Figure 3 shows the XRD patterns of the TiO_2_-CNF catalyst supports fabricated with different flow rates. Figure 3a is an individual sample for the catalyst support, which is F0.1 sample, to look the close-up XRD pattern with all the peak material in TiO_2_-CNF, while Fig. 3b is the entire flow rate sample involved. The existence of TiO_2_ and carbon in the sample is featured. The TiO_2_ consists of two structures, anatase and rutile, because the carbonization temperature converts a small amount of anatase TiO_2_ into rutile TiO_2_. The diffraction peaks for anatase TiO_2_ are 25° (101), 38° (112), 48° (200), 53.9° (105), 62° (213), and 68° (116), while those for rutile, TiO_2_ are 27° (110), 36° (101), 41° (111), and 54° (211). The carbon source is indicated by several diffraction peaks, including those at 30° (110) and 55° (211). The anatase and rutile TiO_2_ formed a tetragonal structure, while carbon was in the face-centered cubic crystallographic structure.

**Fig. 4:**
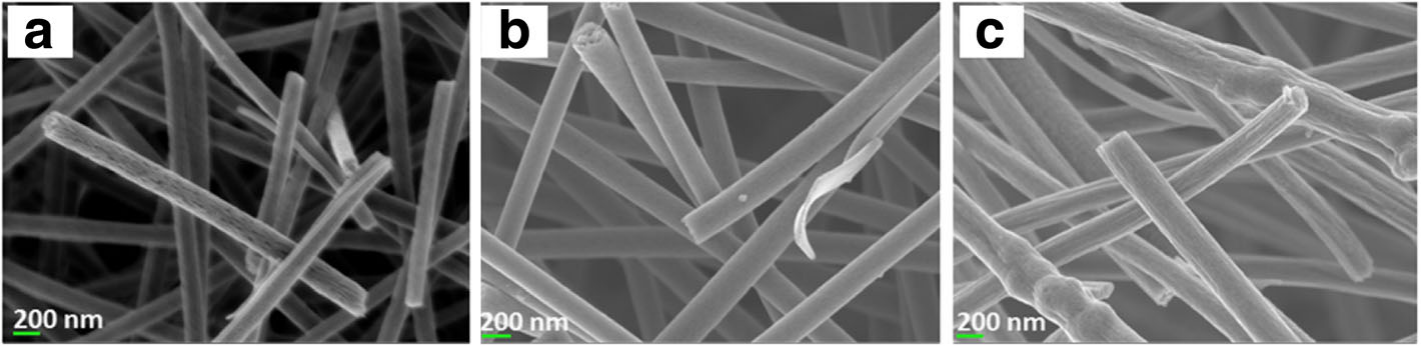
FESEM image of **a** TiO_2_-CNF (F0.1), **b** TiO_2_-CNF (F0.5), and **c** TiO_2_-CNF (F0.9) at ×30,000 magnification

**Fig. 5:**
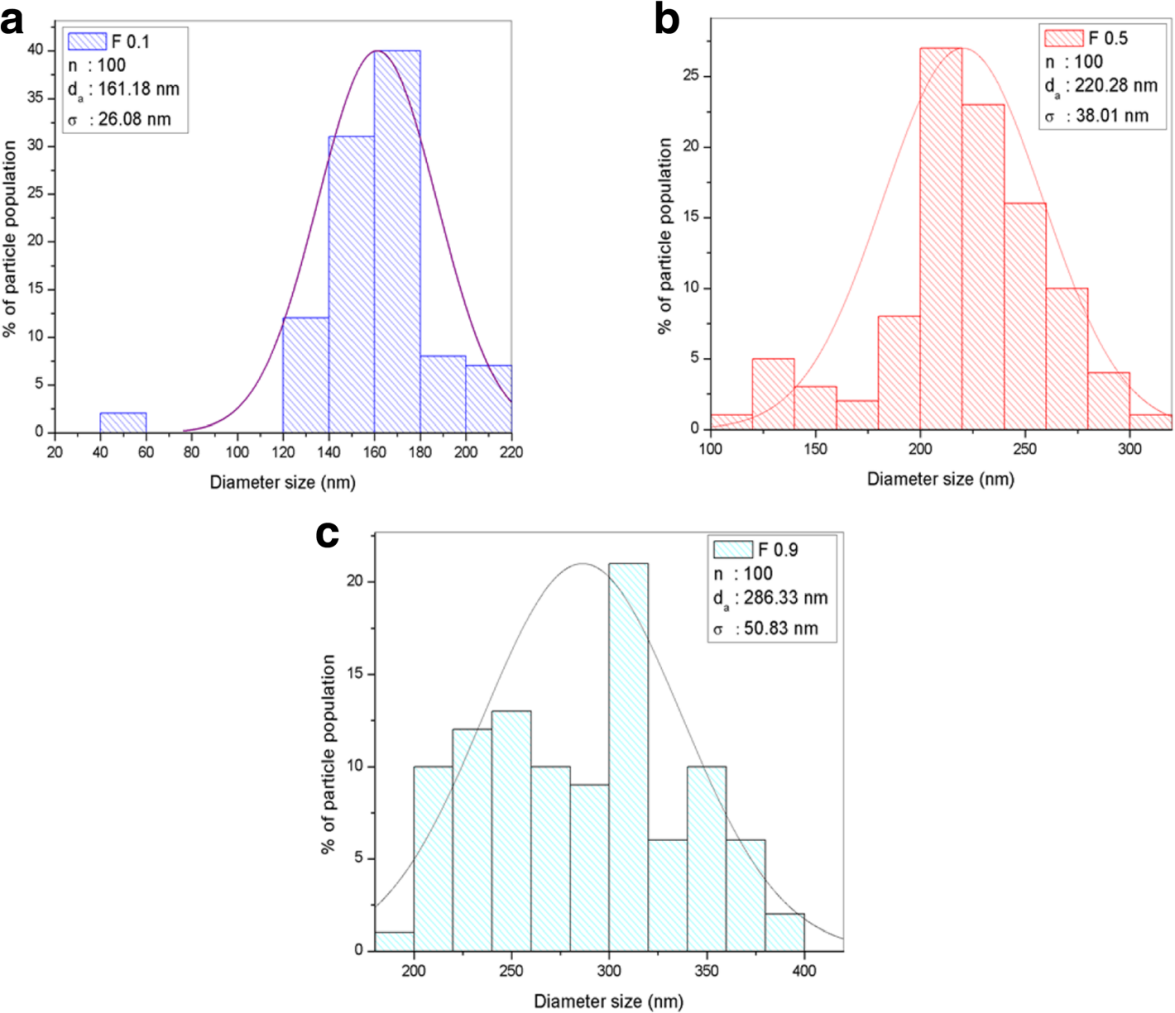
Histogram data of diameter size distribution with the parameter of n, d_a_, and σ. **a** TiO_2_-CNF (F0.1). **b** TiO_2_-CNF (F0.5). **c** TiO_2_-CNF (F0.9)

The flow rate in the electrospinning technique was found to affect the nanofiber diameter, which was calculated using FESEM analysis. The FESEM image is presented in Fig. 4, while the results of the size distribution and diameter are shown in Fig. 5 and Table 1. The image confirms that the nanofiber structure was formed at all studied flow rates. The F0.1 nanofibers showed a smooth morphology due to the slower flow rate, which gives enough time for the solvent to evaporate, helping to produce the smooth structure. The mean nanofiber diameter from 100 measurements is 161.18 ± 26.08 nm, which is the smallest diameter among the samples produced at different flow rates. However, the FESEM image of F0.5 shows the formation of flat ribbons on the nanofibers due to the lack of evaporation from the core, i.e., the solvent is entrapped in the core and diffuses to the ambient atmosphere to cause the flat ribbon structure [21]. F0.9 shows more rough nanofibers with non-uniform diameters, and several beads formed on the nanofiber morphology. This occurs when the flow rate is much higher than the optimum value, which reduces the drying time before the fiber reaches the collector. The mean nanofiber diameters of F0.5 and F0.9 were higher than that of F0.1, which were 220.28 ± 38.01 and 286.33 ± 50.83 nm, respectively. The FESEM image reveals that the diameter of the nanofibers increases as the flow rate increases during electrospinning. F0.1, which has a flow rate of 0.1 mLhr^−1^, was used for further analysis on the effect of DTC on the nanofiber diameter.

**Table 1:** The diameter size distribution of nanofiber for all the sample with different flow rate

Experiment	Voltage (kV)	Flow rate (mlhr^−1^)	DTC (cm)	*n*	Mean diameter, d_a_ (nm)	Std. deviation, σ (± nm)
F0.1	16	0.1	16	100	161.18	26.08
F0.5	16	0.5	16	100	220.28	38.01
F0.9	16	0.9	16	100	286.33	50.83

#### Effect of the Distance Between the Tip and Collector

**Fig. 6:**
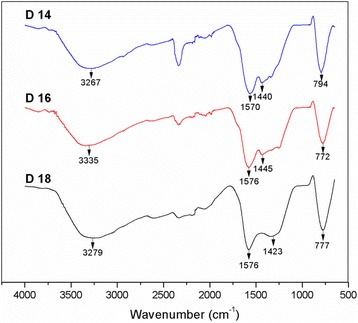
The IR spectrum for the TiO_2_-CNF sample with different DTC parameter at the range of 650 to 4000 cm^−1^ wavenumber

The synthesized TiO_2_-CNF catalyst supports were analyzed by FTIR to evaluate the chemical bonding in the samples, and the IR spectra of the samples are illustrated in Fig. 6. The IR spectra show three samples with different DTC parameters after the carbonization process. All the synthesized samples show the existence of O-Ti-O and carbonate ion bonding, where the peaks and wavenumbers in the spectra were in the same range as those in the F0.1, F0.5, and F0.9 samples in the previous section. The wavenumbers were close enough to indicate the similarity of the samples, including the samples produced at different flow rates in Fig. 2. However, sample D14 shows an existence of a new peak around 2300–2400 cm^−1^, which indicate the N-H stretching vibrations. This peak can be categorized as tertiary amine salts peak, where the N-H bond is weak and of no practical value that can be neglected [19]. The presence of this bond might be due to the incomplete removal of the solvent compound during carbonization process. This observation shows that the electrospinning parameters of flow rate and DTC do not influence the chemical bonding in the samples.

**Fig. 7:**
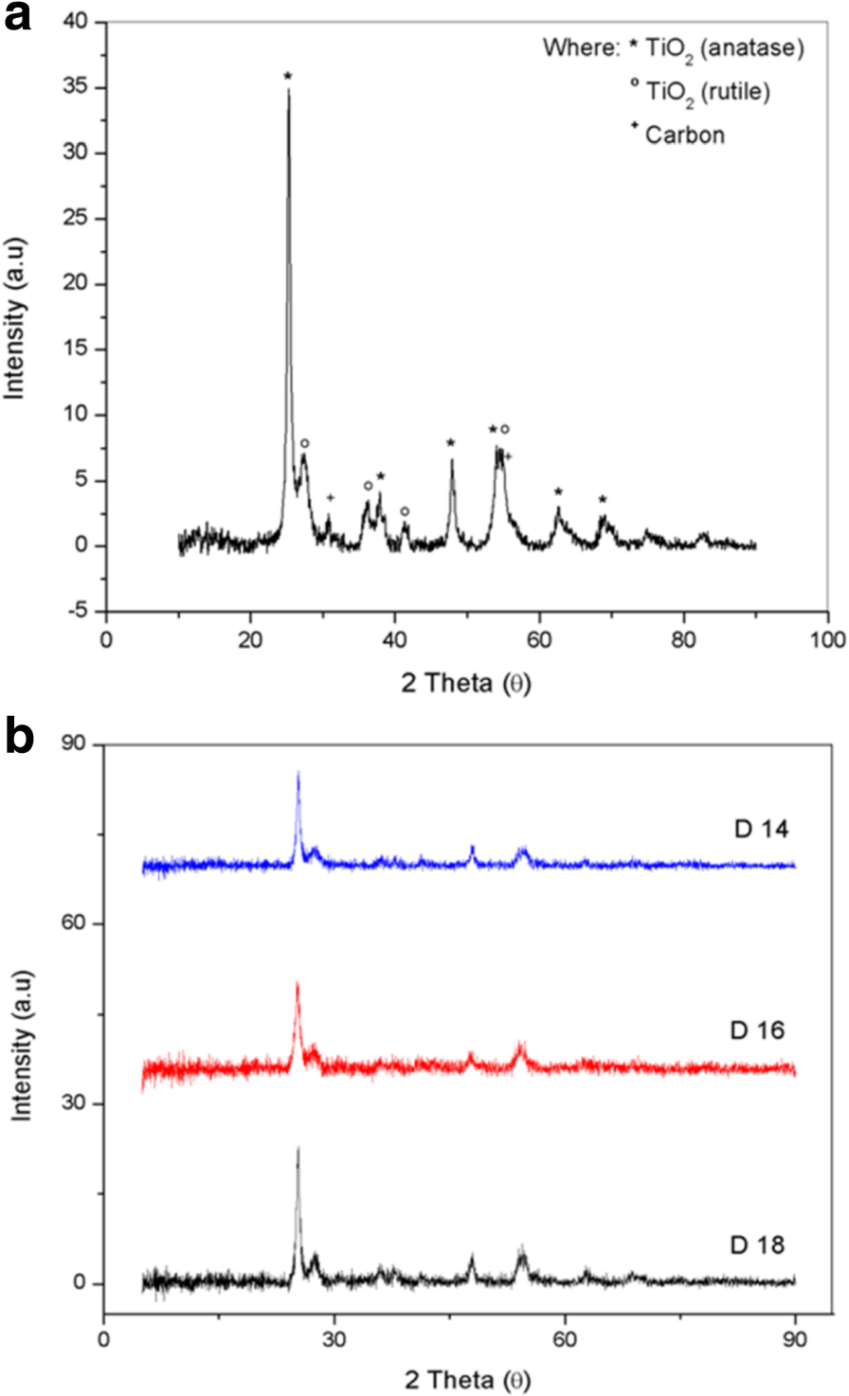
X-ray diffraction patterns of **a** individual TiO_2_-CNF sample and **b** different DTC sample

The crystallinity of the TiO_2_-CNF catalyst supports was analyzed. The XRD patterns are shown in Fig. 7a for individual sample and Fig. 7b for DTC. The individual sample in Fig. 7a indicates the close-up XRD pattern for DTC 18 to see the existence peak for all the material involved. The materials involved in the catalyst support, TiO_2_ and carbon, are shown to exist in each sample. The diffraction peaks were examined over a 2θ range of 5° to 90°, and the peaks at 31° (110) and 55° (211) indicate that carbon with a FCC crystallographic structure is present in the catalyst support. The sharp diffraction peak at 25° (101) was attributed to TiO_2_ in anatase form, and there are several other peaks for anatase TiO_2_, including those at 38° (004), 48° (200), 53° (105), 55° (211), 63° (204), and 69° (116). The other four diffraction peaks at 27° (110), 36° (101), 41° (111), and 54° (211) belong to rutile TiO_2_. Both anatase and rutile TiO_2_ have a tetragonal crystallographic structure.

**Fig. 8:**
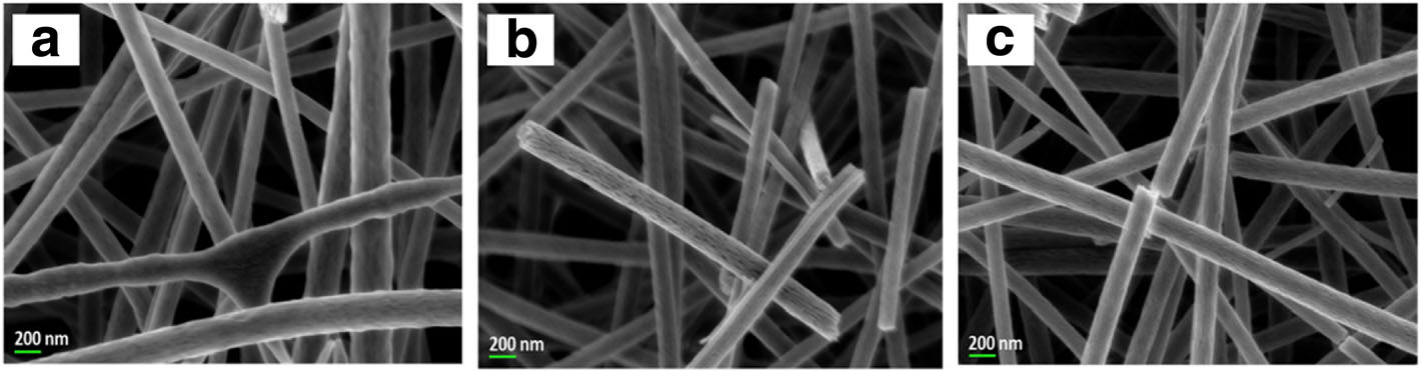
FESEM image of **a** TiO_2_-CNF (D14), **b** TiO_2_-CNF (D16), and **c** TiO_2_-CNF (D18) at ×30,000 magnification

**Fig. 9:**
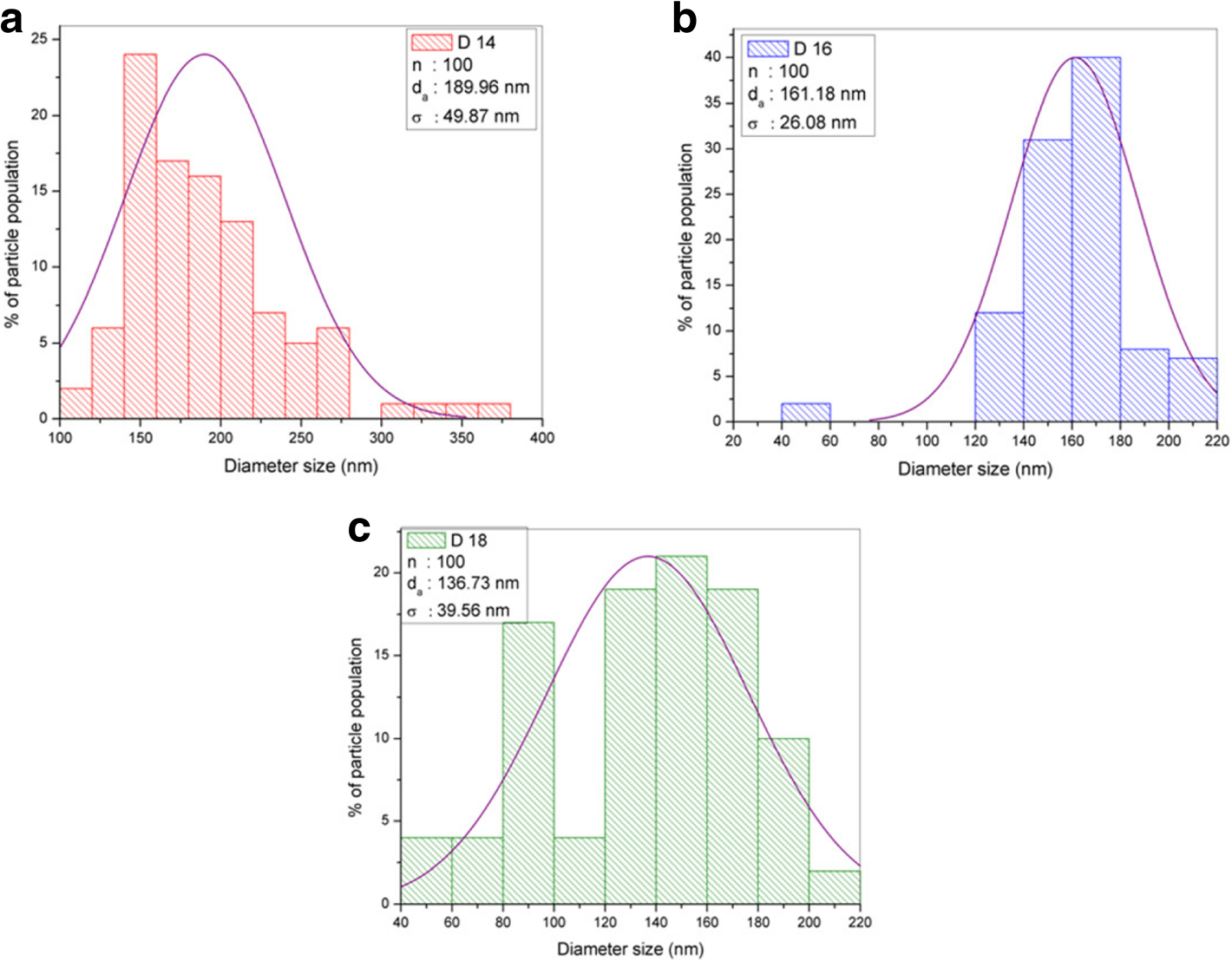
Histogram data of diameter size distribution with the parameter of n, d_a_, and σ. **a** TiO_2_-CNF (D14). **b** TiO_2_-CNF (D16). **c** TiO_2_-CNF (D18)

TiO_2_-CNF nanofibers were fabricated via electrospinning with different DTC values, denoted D14, D16, and D18. The DTC was varied to 14, 16, and 18 cm. The diameter of the nanofiber was calculated using FESEM analysis. Figure 8 shows the FESEM images of the samples with different DTC values at ×30,000 magnification. The effect of the variation in DTC on the diameter of the nanofibers was estimated using the particle size distribution (diameter), as illustrated in Fig. 9, and the values are tabulated in Table 2. The diameter distribution includes several parameters, n (nanoparticle population), d_a_ (arithmetic mean particle size), and σ (standard deviation) [22].

**Table 2:** The diameter size distribution of nanofiber for all the sample with different DTC

Experiment	Voltage (kV)	Flow rate (mlhr^−1^)	DTC (cm)	*n*	Mean diameter, d_a_ (nm)	Std. deviation, σ (± nm)
D14	16	0.1	14	100	189.96	49.87
D16	16	0.1	16	100	161.18	26.08
D18	16	0.1	18	100	136.73	39.56

The smallest mean diameter was 136.73 ± 39.56 nm (90–170 nm), belonging to D18, followed by D16 and D14 with diameters of 161.18 ± 26.08 and 189.96 ± 49.87 nm, respectively. The longer the tip-collector distances, the smaller the nanofiber diameter. This behavior is due to the deposition time and whipping instability interval during the electrospinning process. The longer distance supplies a longer deposition time, and during that period, the whipping instability phenomenon occurs, also known as the thinning and splitting mechanism. This phenomenon occurs due to interactions between charged ions and the electric field [17]. When the electrical force applied to the nozzle tip reaches a critical value, the highly charged density and viscoelastic force split the jets into smaller jets, creating a bending, winding, and spiraling path towards the collector. When the DTC is longer, jet splitting repeatedly occurs, resulting in ultrafine and smaller diameter fibers. Therefore, the smallest diameter belongs to sample D18 with a flow rate of 0.1 mLh^−1^ and DTC of 18.

The diameter of fabricated nanofibers, TiO_2_-CNF, is compared with the previous study of nanofibers diameter for TiO_2_-based nanofibers, and this comparison is shown in Table 3. The results show that the TiO_2_-CNF is having the smallest nanofiber diameter with 136.73 ± 39.56 nm. This is due to the optimum parameter used during electrospinning method; which is low in flow rate and high value of DTC. Thus, with smaller flow rate and higher DTC value, the smaller diameter of a nanofiber produced. This shows that the electrospinning parameters give the highest effect to the diameter of nanofiber. Even though a longer DTC and smaller flow rate are preferable, there are optimum values for these parameters, because these parameters can lead to a loss in weight. This occurs due to over-evaporation, in which the nanofiber forms before reaching the collector, allowing the nanofiber to freely travel to undesired regions.

**Table 3:** Comparison of the nanofiber diameter with the previous study

Authors	Type of nanofiber	Electrospinning parameters		Diameter of nanofiber (nm)
		Flow rate (mLhr^−1^)	DTC (cm)	
This study	TiO_2_-CNF	0.1	18	136.73 ± 39.56
Garcia-Gomez et al. [26]	TiO_2_/PANI/PVP	0.8	13	200
Li et al. [27]	TiO_2_ nanofibers	0.2	5	192 ± 69
Mehrpouya et al. [28]	AC/TiO_2_ NCNFs	0.2	15	244
Mondal et al. [29]	PVP/Ti(OiPr)_4_	2.4	5	210

**Fig. 10:**
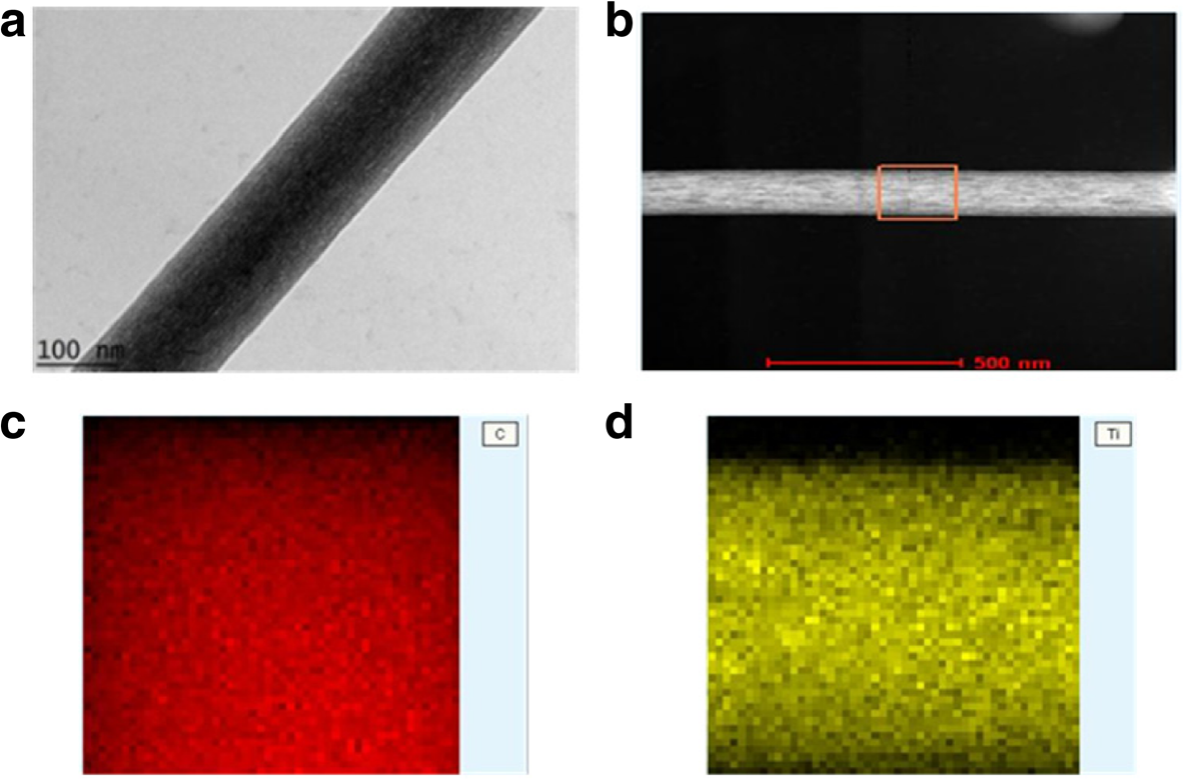
TiO_2_-CNF catalyst support **a** TEM image (scale 100 nm), **b** TEM image for TiO_2_ and C nanofiber mapping, **c** mapping for C nanofiber, and **d** mapping for TiO_2_ nanofiber

The D18 sample with a flow rate of 0.1 mLh^−1^ and DTC of 18 was selected for TEM analysis to examine the morphology and obtain the diameter size. The TEM image and elemental mapping of the TiO_2_-CNF catalyst support are shown in Fig. 10. The TEM image shows that TiO_2_-CNF results in smooth and silky nanofiber with a diameter of 135.38 nm. The diameter is in the same range (90–170 nm) as that obtained from FESEM analysis. Mapping is employed to examine the distribution of TiO_2_ and carbon on nanofiber. The results reflect that TiO_2_ and carbon formed uniformly in the nanofiber structure, due to the homogeneous distribution of the polymer solution and the TiO_2_ precursor during the sol-gel method. This mapping also shows the location of the materials, in which TiO_2_ and carbon are located along the entire nanofiber surface, which benefits the creation of active reaction areas during catalysis. The other nanofiber samples are expected to have the same even distribution of TiO_2_ and carbon. The particle size of TiO_2_ and carbon in the nanofiber samples and their effect towards MOR is discussed in the next section.

### Electrochemical Characterization of the Methanol Oxidation Reaction

**Fig. 11:**
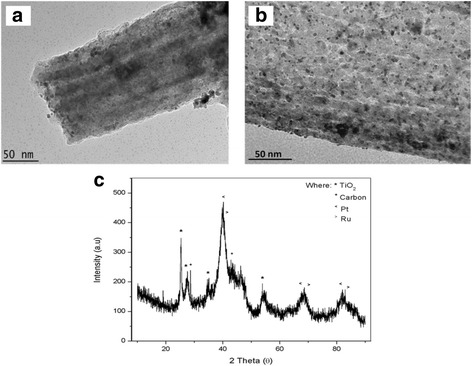
Image of PtRu deposited on TiO_2_-CNF **a** TEM image of PtRu/TiO_2_-CNF in F0.1, **b** TEM image of PtRu/TiO_2_-CNF in D18, and **c** XRD pattern of PtRu/TiO_2_-CNF of D18

Electrochemical characterization involves three main steps, which are characterization of the electrochemical activity, the electrocatalytic performance, and the long-term stability and durability. The electrochemical activity and electrocatalytic performance were analyzed by CV using a three-electrode system to acquire qualitative and quantitative information about the involved reaction [23]. Figure 11a, b shows the TEM image of the PtRu catalyst deposited on the surface of the F0.1 and D18 TiO_2_-CNF supports, respectively. The PtRu catalyst was evenly distributed on the surface of nanofibers in both F0.1 and D18. Figure 11c shows the XRD pattern of D18 PtRu/TiO_2_-CNF, while Table 4 gives the data for the nanofiber diameter, obtained from FESEM, and crystallite size of the particles in the samples, obtained from XRD. Table 4 shows that the F series samples (F0.1, F0.5, and F0.9) with added catalyst have a TiO_2_ (anatase) crystallite size of approximately 20 to 22 nm. The change in nanofiber diameter has little effect on the crystallite size of TiO_2_, while the crystallite size of carbon changes as the nanofiber diameter increases from 15.9 nm in F0.1 to 25.8 nm in F0.9. The crystallite size of Pt also tends to increase with the carbon crystallite size. The crystallite size of Pt supported on F0.1, F0.5, and F0.9 is 5.67, 8.04, and 9.75 nm, respectively. The changes in the Pt crystallite size are due to the surface properties of the nanofiber. Table 4 also shows the crystallite size of PtRu supported on D series samples. The nanofiber diameter decreases in value from D14 to D16 to D18. In contrast to the F series samples, the crystallite size of TiO_2_ (anatase) in the D series samples decreases as the nanofiber diameter decreases. The TiO_2_ crystallite size is 23.40, 21.50, and 18.60 nm for D14, D16, and D18, respectively. The crystallite size of carbon and Pt also decreases with a decrease in the nanofiber diameter. The carbon crystallite size decreases from 17.3 nm in D14 to 14.4 nm in D18, while the Pt crystallite size supported on D14, D16, and D18 is 5.44, 5.67, and 4.64 nm, respectively. From these data, the changes in the crystallite sizes of TiO_2_ and carbon in the nanofiber lead to changes in the surface properties of the nanofiber, leading to the changes in the crystallite size of the Pt particles deposited on the surface of the nanofiber.

**Table 4:** Diameter of nanofiber from FESEM and crystallite size of particle in catalyst from XRD data

Catalyst	Average diameter of nanofiber from FESEM	Crystallite size (nm)		
		TiO_2_	C	PtRu
PtRu/TiO_2_-CNF (F0.1)	161.18	21.50	15.9	5.67
PtRu/TiO_2_-CNF (F0.5)	220.28	22.60	16.5	8.04
PtRu/TiO_2_-CNF (F0.9)	286.33	20.36	25.8	9.75
PtRu/TiO_2_-CNF (D14)	189.96	23.40	17.3	5.44
PtRu/TiO_2_-CNF (D16)	161.18	21.50	15.9	5.67
PtRu/TiO_2_-CNF (D18)	136.73	18.60	14.4	4.64

**Fig. 12:**
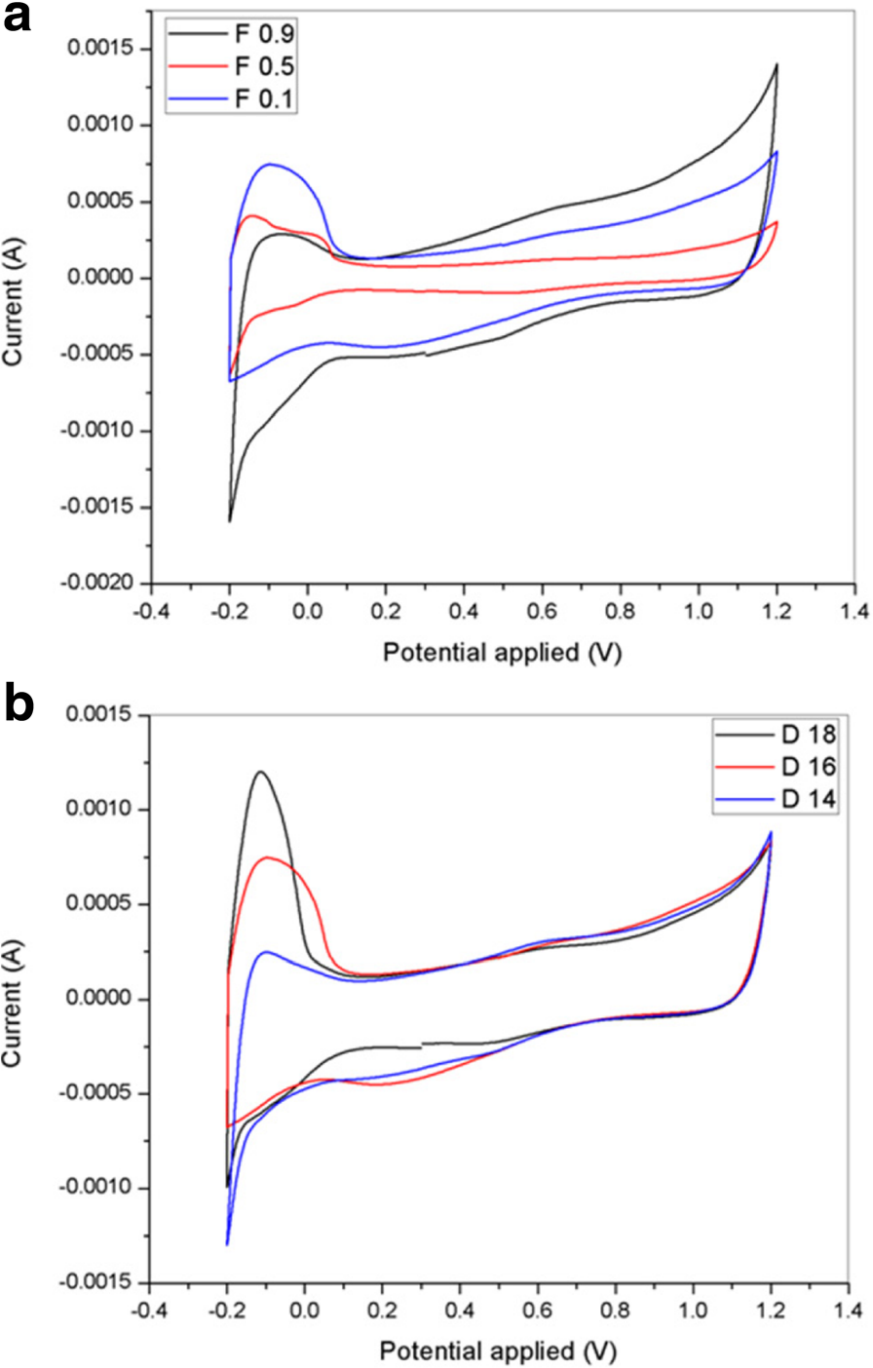
Cyclic voltammetry profiles of the PtRu/TiO_2_-CNF with **a** different flow rate, F0.1, F0.5, F0.9, and **b** different DTC, D14, D16, and D18, in 0.5 M H_2_SO_4_ solution at the scan rate of 50 mVs^−1^

Figure 12 shows the CV profiles of the PtRu/TiO_2_-CNF electrocatalysts with different catalyst supports in 0.5 M H_2_SO_4_ solution. The CV curve for F0.1, F0.5, and F0.9 are shown in Fig. 12a, while D14, D16, and D18 are shown in Fig. 12b. Hydrogen adsorption-desorption by Pt occurs around − 0.2 to 0.1 V vs. Ag/AgCl. The mass loading for all the electrocatalyst in this profile is the same that as 0.57 mgcm^−2^. The PtRu/TiO_2_-CNF supported on D18 exhibits a steep current peak for hydrogen adsorption in comparison in the other D series samples, while F0.1 has a steep peak in comparison with the F series samples. The peak indicates that the active surface area on the PtRu/TiO_2_-CNF electrocatalyst and the ECSA can be calculated from the equation: ECSA = *Q*/(*Γ*.*W*_Pt_). Where, Q is the integral of the hydrogen adsorption area, Γ is the constant for the charge required to reduce the proton monolayer on the Pt (2.1 Cm_Pt_^−2^), and *W*_Pt_ is the mass loading of Pt. Table 5 shows the ECSA of all the catalyst samples in units of m^2^ g^−1^ with mass loadings according to the mass of PtRu. From Table 5, the ECSA for PtRu supported on F0.1, F0.5, and F0.9 is 131.29, 65.05, and 25.03 m^2^ g^−1^, respectively. The ECSA value decreases with increasing Pt crystallite size in the catalyst samples. The catalyst supported on D14, D16, and D18 has an ECSA value of 21.48, 131.29, and 226.75 m^2^ g^−1^, respectively. As shown previously, the value of the Pt crystallite size in the D series samples decreases from D14 to D18, and thus, the ECSA increases according to Pt crystallite size. Smaller size particles lead to an increase in the active surface area of the catalyst. Overall, the electrospinning parameters clearly show big influence towards the diameter and surface properties (surface morphology) of nanofibers.

**Table 5:** Summary of the peak potential, current density, CO tolerance, and ECSA results for the catalyst with the different electrospinning parameter

Catalyst	ECSA, (m^2^g^−1^_PtRu_)	Peak potential, (V vs. Ag/AgCl)	Onset potential, (V vs. Ag/AgCl)	Peak current density, (mAmg^−1^_PtRu_)	CO tolerance, I_f_/I_b_ ratio
PtRu/C	16.94	0.722	0.398	79.32	1.92
PtRu/TiO_2_-CNF (F0.1)	131.29	0.771	0.361	249.58	3.82
PtRu/TiO_2_-CNF (F0.5)	65.05	0.769	0.375	225.83	2.06
PtRu/TiO_2_-CNF (F0.9)	25.03	0.635	0.334	205.89	5.61
PtRu/TiO_2_-CNF (D14)	21.48	0.754	0.363	201.45	2.16
PtRu/TiO_2_-CNF (D16)	131.29	0.771	0.361	249.58	3.82
PtRu/TiO_2_-CNF (D18)	226.75	0.732	0.366	274.72	3.81

**Fig. 13:**
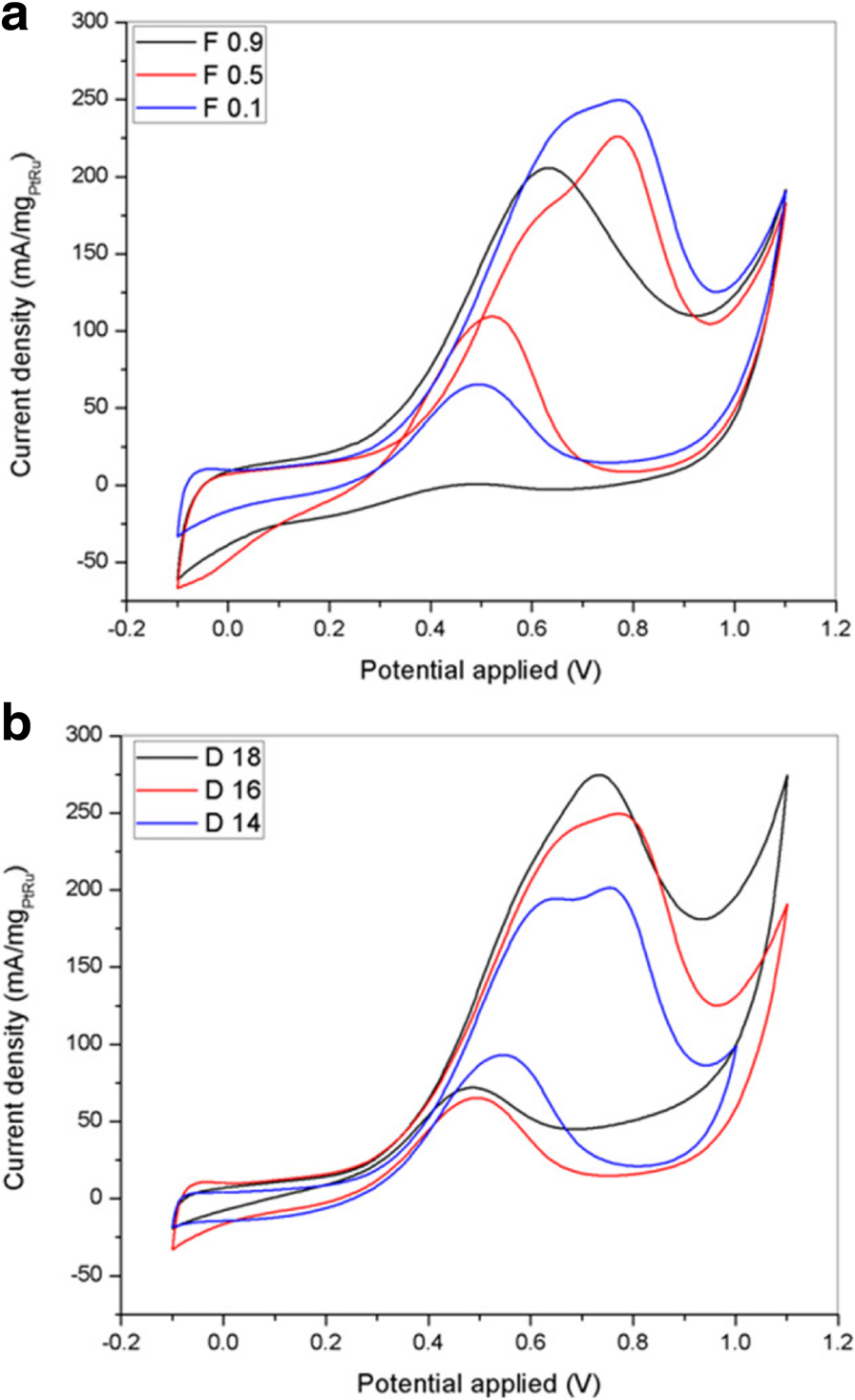
Cyclic voltammograms for PtRu/TiO_2_-CNF with different **a** flow rate and **b** DTC of the catalyst support in 2 M methanol and 0.5 M H_2_SO_4_ at the scan rate of 50 mVs^−1^

The electrocatalytic performance of PtRu supported on the different F and D series nanofibers is tabulated and plotted in Table 5 and Fig. 13. The CV curve was measured in 2 M methanol and 0.5 M H_2_SO_4_ solution saturated with N_2_ gas at room temperature. The mass loading for all the electrocatalyst is the same which is 0.57 mgcm^−2^. Figure 13 shows multiple CV curves over a potential range of − 0.1 to 1.1 V vs. Ag/AgCl. Figure 13a shows the CV graphs for PtRu supported on the F series nanofiber samples. As the diameter of the nanofiber decreases from sample F0.9 to F0.1, the current density in MOR increases, and the oxidation peak and onset potential of MOR shift towards positive values. On the other hand, in the D series nanofiber samples, the oxidation peak potential of the catalyst supported on D14, D16, and D18 is 0.754, 0.771, and 0.732 V (vs. Ag/AgCl), respectively. There is no pattern in the oxidation peak potential in the D series samples, and the onset potential is almost the same for each sample, at 0.36 V vs. Ag/AgCl. However, the peak current density at the oxidation peak potential of MOR increases in accordance to the catalyst support on D14, D16, and D18. The peak current density for D14, D16, and D18 is 201.45, 249.58_,_ and 274.72 mAmg^−1^_PtRu_, respectively. It can be clearly seen that the increase in the current density matches the patterns in the diameter, from FESEM analysis, and ECSA value. This shows that a smaller diameter size produces high surface area and increases the number of active sites on the electrocatalyst surface. The higher peak current for the composite electrocatalyst may result from the supporting material (TiO_2_-CNF), where changes in the structure and the combination of materials can be very effective in producing positive effects on the metal-support interaction [5, 24].

The reverse scan in the CV curve shows a small oxidation peak at a potential of approximately 0.49–0.55 V vs. Ag/AgCl. This second oxidation peak appeared due to the incomplete removal of oxidized carbonaceous species in the forward scan [25]. However, the ratio between the forward (I_f_) and reversed (I_b_) oxidation peak for PtRu/TiO_2_-CNF (D18) exceeded 3.8, which means that the electrocatalyst has high tolerance towards carbonaceous species, reducing the potential for catalyst poisoning. This result shows that the combination of metal oxide and carbon nanofibers has a good potential for use in fuel cell applications.

## Conclusion

TiO_2_-CNF nanofibers can be fabricated via electrospinning, which is the main technique, and several other methods. The nanofibers are influenced by the flow rate and the DTC, which were examined as electrospinning process parameters, with three different samples for each parameter, denoted F0.1, F0.5, F0.9, D14, D16, and D18. The results showed that the TiO_2_-CNF (D18) sample produced the smallest average diameter of 136.73 ± 39.56 nm. TiO_2_-CNF was mixed with PtRu to form the composite catalyst, and its CV performance was examined. The current density of the PtRu/TiO_2_-CNF (D18) sample is 1.4 times higher than that of PtRu/TiO_2_-CNF (D14), while the ECSA of PtRu/TiO_2_-CNF (D18) is 10 times higher than that of the other samples. Thus, the flow rate and DTC highly affect the diameter, morphology, and performance of the nanofibers. The nanofiber performance increased with decreasing nanofiber diameter, which shows the capability of the composite nanofiber catalyst to be an upcoming anode catalyst for DMFCs.
